# Phospholipid Arachidonic Acid Remodeling During Phagocytosis in Mouse Peritoneal Macrophages

**DOI:** 10.3390/biomedicines8080274

**Published:** 2020-08-05

**Authors:** Luis Gil-de-Gómez, Patricia Monge, Juan P. Rodríguez, Alma M. Astudillo, María A. Balboa, Jesús Balsinde

**Affiliations:** 1 Instituto de Biología y Genética Molecular, Consejo Superior de Investigaciones Científicas (CSIC), Universidad de Valladolid, 47003 Valladolid, Spain; gildegomel@email.chop.edu (L.G.-d.-G.); pmonge@ibgm.uva.es (P.M.); rodriguezcasco@me.com (J.P.R.); alma@ibgm.uva.es (A.M.A.); mbalboa@ibgm.uva.es (M.A.B.); 2 Centro de Investigación Biomédica en Red de Diabetes y Enfermedades Metabólicas Asociadas (CIBERDEM), 28029 Madrid, Spain; 3 Laboratorio de Investigaciones Bioquímicas de la Facultad de Medicina (LIBIM), Instituto de Química Básica y Aplicada del Nordeste Argentino (IQUIBA-NEA), Universidad Nacional del Nordeste, Consejo Nacional de Investigaciones Científicas y Técnicas (UNNE-CONICET), Corrientes 3400, Argentina

**Keywords:** arachidonic acid, eicosanoids, phospholipid remodeling, phospholipase A_2_, inflammation, monocytes/macrophages

## Abstract

Macrophages contain large amounts of arachidonic acid (AA), which distributes differentially across membrane phospholipids. This is largely due to the action of coenzyme A-independent transacylase (CoA-IT), which transfers the AA primarily from diacyl choline-containing phospholipids to ethanolamine-containing phospholipids. In this work we have comparatively analyzed glycerophospholipid changes leading to AA mobilization in mouse peritoneal macrophages responding to either zymosan or serum-opsonized zymosan (OpZ). These two phagocytic stimuli promote the cytosolic phospholipase A_2_-dependent mobilization of AA by activating distinct surface receptors. Application of mass spectrometry-based lipid profiling to identify changes in AA-containing phospholipids during macrophage exposure to both stimuli revealed significant decreases in the levels of all major choline phospholipid molecular species and a major phosphatidylinositol species. Importantly, while no changes in ethanolamine phospholipid species were detected on stimulation with zymosan, significant decreases in these species were observed when OpZ was used. Analyses of CoA-IT-mediated AA remodeling revealed that the process occurred faster in the zymosan-stimulated cells compared with OpZ-stimulated cells. Pharmacological inhibition of CoA-IT strongly blunted AA release in response to zymosan but had only a moderate effect on the OpZ-mediated response. These results suggest a hitherto undescribed receptor-dependent role for CoA-independent AA remodeling reactions in modulating the eicosanoid biosynthetic response of macrophages. Our data help define novel targets within the AA remodeling pathway with potential use to control lipid mediator formation

## 1. Introduction

Macrophages play a key role in host defense against external aggression by sensing the cellular microenvironment from strategic positions in all tissues. When a foreign agent is detected, the macrophages phagocytize and process it, and present antigens to T cells in order to initiate the adaptive immune response [1,2]. The initial recognition process leading to phagocytosis involves the participation of a wide variety of membrane receptors, collectively called opsonin-independent receptors, which include C-type lectins such as dectin-1, the mannose receptor, scavenger receptors, and Toll-like receptors. Additionally, macrophages are endowed with opsonin-dependent receptors that recognize the Fc fragment of immunoglobulins (IgG) and complement fractions (C3b, C4b) that coat the target as opsonins, and facilitate the intake process [3].

Once the target, either native or opsonized, has been detected, phagocytic receptors undergo an oligomerization process and contact structural elements of the cellular cytoskeleton, which enables the macrophages to gather information about the bodily nature of a target. Next, a series of intracellular remodeling processes occur, including actin polymerization, that support membrane pseudopod extension leading to the formation of a phagosome. Several lipid cell signaling mechanisms have been described in this step, such as the phosphorylation of phosphatidylinositol-4,5-bisphosphate by phosphoinositide-3-kinase to produce phosphatidylinositol-3,4,5- trisphosphate, and the enrichment of the outer leaflet of the plasmalemma in sphingolipids to foster F-actin accumulation in the pseudopod [4,5], as well as the mobilization of large amounts of free arachidonic acid (AA) for the synthesis of bioactive eicosanoids [6,7].

Zymosan particles, prepared from cell walls of the yeast *S. cerevisiae*, have been extensively used as a model to study innate immune responses to fungal infections [4,5,8]. Zymosan particles interact with multiple receptors on the surface of macrophages, including dectin-1 and Toll-like receptors 2 and 6, which mediate internalization of the particle and the activation of transcriptional events [4,5,8]. Interestingly, studies utilizing dectin-1-deficient mice have demonstrated that dectin-1 is the receptor that specifically and selectively couples fungal responses to enhanced AA release and eicosanoid production in innate immune cells [9,10]. On the other hand, serum-opsonized zymosan (OpZ), coated with IgG and complement factors, is also an excellent stimulus for AA release and eicosanoid production and, in this case, the process is mediated by engagement of Fc receptors [11]. Although receptors for complement, particularly CR3, actively participate in the recognition and uptake of OpZ, it has been demonstrated that engagement of these receptors is not coupled to phospholipase A_2_ (PLA_2_) activation and AA mobilization [9,10,11,12]. Thus, depending on the nature of the stimulus, native or opsonized zymosan, two different innate immune receptors, dectin-1 or FcRs, mediate the eicosanoid response.

Both dectin-1 and FcRs share a number of commonalities with regard to their mechanisms of cellular activation and signal transduction, but also exhibit marked differences. They signal via immunoreceptor tyrosine-based activation motifs located in the receptor tail, and involve tyrosine phosphorylation cascades controlled by Src family kinases or CD45 and CD148 phosphatases. However, Fc receptor-mediated phagocytosis manifests a strong dependence on Syk activation, while dectin-1-mediated phagocytosis does not [13,14].

While the stages of contact, internalization, and activation of transcriptional programs by native or opsonized antigens are well described, much lesser attention has been given to the phospholipid fatty acid remodeling processes occurring during phagocytosis. In previous work from our laboratory, we demonstrated the involvement of several PLA_2_ forms, each acting on distinct phospholipid pools and releasing different fatty acids, during zymosan stimulation of mouse peritoneal macrophages [15,16,17]. In addition, we showed that hydrolysis of ethanolamine-containing phospholipids at the membrane is a key event to support phagocytosis of zymosan and live bacteria by human macrophages [18]. Studies in murine macrophages confirmed that cells deficient in membrane ethanolamine plasmalogens show reduced capacity to phagocytize opsonized zymosan particles. Overcoming such deficiency by incubating the macrophages with lysoplasmalogen significantly restores their phagocytic capacity [19,20].

To further explore the involvement of membrane phospholipid remodeling during phagocytosis, in this work, we have applied mass spectrometry-based lipidomic approaches to identify changes in AA-containing phospholipids in mouse peritoneal macrophages exposed to either zymosan or OpZ. Both stimuli induce a rapid release of AA to the extracellular medium, part of which is converted into a number of eicosanoids. While the time- and dose-dependence of the effects are similar for both stimuli, the response to OpZ was consistently higher than to zymosan. These distinct features appear to be related to the differential involvement of phospholipid remodeling pathways catalyzed by coenzyme A-independent transacylase (CoA-IT), which mediates the transfer of AA moieties between various phospholipid species.

## 2. Materials and Methods 

### 2.1. Reagents

Cell culture medium was from Molecular Probes-Invitrogen (Carlsbad, CA, USA). Organic solvents (Optima^®^ LC/MS grade) were from Fisher Scientific (Madrid, Spain). Lipid standards were from Avanti (Alabaster, AL, USA) or Cayman (Ann Arbor, MI, USA). Silicagel G thin-layer chromatography plates were from Macherey-Nagel (Düren, Germany). [5,6,8,9,11,12,14,15-^3^H]Arachidonic acid (180 Ci/mmol) was from PerkinElmer (Boston, MA, USA). The PLA_2_ inhibitors pyrrophenone and FKGK18 were from Cayman. The CoA-independent transacylase inhibitor SKF98625 (diethyl 7-(3,4,5-triphenyl-2-oxo-2,3-dihydroimidazole-1-yl)heptane phosphonate) [21,22,23] was synthesized and provided by Dr. A. Pérez (Department of Organic Chemistry, University of Valladolid). All other reagents were from Sigma-Aldrich (Madrid, Spain).

### 2.2. Cell Culture

Primary cultures of resident peritoneal macrophages (Swiss male mice, 10–12 weeks old, University of Valladolid Animal House) were established as described previously [24]. The cells were left to adhere to plastic culture dishes by incubating them at 37 °C for 20 h in a humidified atmosphere at 5% CO_2_ and 95% air in Roswell Park Memorial Institute 1640 medium with 10% (*v/v*) fetal bovine serum, 100 U/mL penicillin, and 100 µg/mL streptomycin, and 2 mM L-glutamine. All procedures involving animals were carried out under the supervision of the Institutional Committee of Animal Care and Usage of the University of Valladolid (Approval No. 7406000), and are in accordance with the guidelines established by the Spanish Ministry of Agriculture, Food, and Environment and the European Union.

For experiments, the cells were placed in serum-free medium for 1 h. Afterward, they were challenged by the stimuli for the times indicated. When inhibitors were used, they were added for 15–30 min before the addition of stimuli. Zymosan was prepared as described [25]. For preparation of OpZ, the particles were treated with heat-inactivated mouse serum (3 mg zymosan per 1 mL serum) for 20 min at 37 °C. As heating the serum inactivates complement factors, the OpZ produced in this manner promotes IgG-mediated responses [26]. Zymosan aliquots were diluted in serum-free medium and sonicated before addition to the cells. No phospholipase A_2_ activity was detected in the zymosan batches used in this study, as assessed by in vitro activity assay [27,28,29,30,31]. A commercial kit based on the Bradford procedure [32] was used to measure cell protein content (BioRad Protein Assay, Bio-Rad, Hercules, CA, USA). To obtain cells labeled with [^3^H]AA, the radioactive fatty acid was added at 0.25 µCi/mL for the 20 h adherence period. Extensive washing was carried out afterward with serum-free medium containing 0.5 mg/mL bovine serum albumin to remove the [^3^H]fatty acid that did not incorporate into cellular lipids. These [^3^H]AA-labeled cells were used for AA release experiments, and the incubations were performed in the presence of 0.5 mg/mL bovine serum albumin [33,34,35,36]. Briefly, after incubation with the stimuli, the supernatants were removed, cleared of detached cells by centrifugation and assayed for radioactivity by liquid scintillation counting.

### 2.3. Measurement of Phospholipid Arachidonate Remodeling

This was carried out exactly as described by Balsinde [37]. Briefly, the cells were pulse labeled with 1 nM [^3^H]AA (0.25 µCi/mL) for 15 min at 37 °C. The cells were then washed four times with medium containing 0.5 mg/mL bovine serum albumin to remove the non-incorporated label. Afterward, the cells were placed in serum-free medium and incubated at 37 °C in the absence or presence of stimuli. After the indicated periods of time, the supernatants were removed and total cellular lipids were extracted according to Bligh and Dyer [38], and separated by thin-layer chromatography with *n*-hexane/diethyl ether/acetic acid (70:30:1, *v/v/v*) [39]. For phospholipid classes, the lipid extracts were separated twice with chloroform/methanol/28% (*w/w*) ammonium hydroxide (60:37.5:4, *v/v/v*) as the mobile phase, using plates impregnated with boric acid [40]. The spots corresponding to each phospholipid class were cut out and assayed for radioactivity by liquid scintillation counting.

### 2.4. Gas Chromatography/Mass Spectrometry (GC/MS) Analyses

Total lipids from approximately 10^7^ cells were extracted according to Bligh and Dyer [38]. After addition of internal standards, lipids were separated by thin-layer chromatography, as described in the preceding paragraph. The bands corresponding to the different lipid classes were scraped off from the plate, and fatty acid methyl esters were obtained from the various lipid fractions by transmethylation with 0.5 M KOH in methanol for 60 min at 37 °C [41,42,43]. Analysis was carried out using an Agilent 7890A gas chromatograph coupled to an Agilent 5975C mass-selective detector operated in electron impact mode (EI, 70 eV), equipped with an Agilent 7693 autosampler and an Agilent DB23 column (60 m length × 0.25 mm internal diameter × 0.15 µm film thickness) (Agilent Technologies, Santa Clara, CA, USA). Data analysis was carried out with the Agilent G1701EA MSD Productivity Chemstation software, revision E.02.00 [41,42,43].

### 2.5. Liquid Chromatography/Mass Spectrometry (LC/MS) Analyses of Phospholipids

This was carried out exactly as described elsewhere [15,16,17,18,19,20,44,45,46,47,48], using a high-performance liquid chromatograph equipped with a binary pump Hitachi LaChrom Elite L-2130 and a Hitachi Autosampler L-2200 (Merck, Madrid. Spain), coupled online to a Bruker Esquire 6000 ion-trap mass spectrometer (Bruker Daltonics, Bremen, Germany). Phospholipid molecular species were identified by multiple reaction monitoring experiments on chromatographic effluent by comparison with previously published data [15,16,17,18,19,20,44,45,46,47,48].

### 2.6. Liquid Chromatography/Mass Spectrometry (LC/MS) Analyses of Eicosanoids

Analysis of eicosanoids by LC/MS was carried out exactly as described elsewhere [15,16,49,50], using an Agilent 1260 Infinity high-performance liquid chromatograph equipped with an Agilent G1311C quaternary pump and an Agilent G1329B Autosampler, coupled to an API2000 triple quadrupole mass spectrometer (Applied Biosystems, Carlsbad, CA, USA). Quantification was carried out by integrating the chromatographic peaks of each species and comparing with an external calibration curve made with analytical standards [15,18,49,50].

### 2.7. Data Analysis

The results are shown as means ± S.E.M. Two groups were compared using the Student’s *t*-test. For more than two groups, one-way analysis of variance (ANOVA) was used, followed by Tukey’s post hoc test. All analyses used GraphPad Prism 8.0 software. *p* < 0.05 was considered significant.

## 3. Results

Yeast-derived zymosan has been used for years as a model to study PLA_2_-dependent pathways for lipid mediator production in murine macrophages [51,52,53,54,55]. Conversely, OpZ has been used most often to stimulate circulating cells such as monocytes and neutrophils [11,56,57,58]. Zymosan activates PLA_2_ via engagement of dectin-1 receptors [9,10], while heat-inactivated OpZ does it by engaging Fc receptors [11,12].

**Figure 1:**
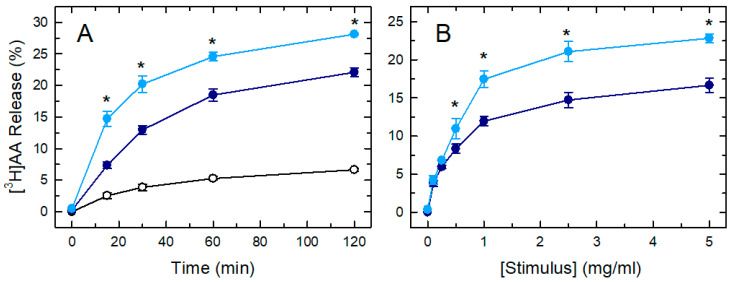
Comparative analysis of the [^3^H]arachidonic acid ([^3^H]AA) release response of mouse peritoneal macrophages responding to either zymosan or serum-opsonized zymosan (OpZ). The cells, prelabeled with [^3^H]AA, were either untreated (open symbols) or treated with zymosan (dark blue) or OpZ (light blue). (**A**) Time-course of [^3^H]AA release (both stimuli added at 1 mg/mL). (**B**) Concentration response curves (1 h incubation). Afterward, the extracellular media were removed and analyzed for ^3^H-radioactivity content. The data are expressed as mean values ± S.E.M. (*n* = 6). * *p* < 0.05, significance of OpZ-stimulated cells versus zymosan-stimulated cells.

Figure 1 shows a comparative analysis of the AA release response of mouse peritoneal macrophages to either zymosan or OpZ. The cells responded to both stimuli by rapidly releasing [^3^H]AA to the extracellular medium (Figure 1A). Maximal effects were observed at zymosan or OpZ concentrations of 1 mg/mL (Figure 1B). While the time- and concentration-dependence of the effects of both zymosan and OpZ on [^3^H]AA release were similar, the response to OpZ was consistently higher than to zymosan.

**Figure 2:**
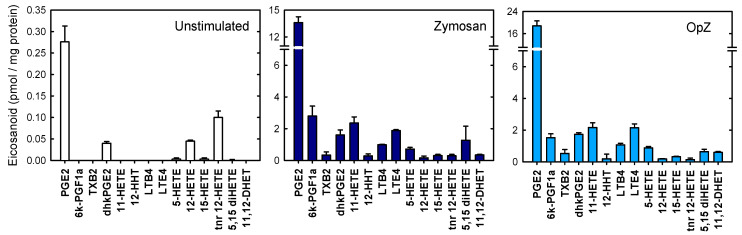
Eicosanoid production by macrophages. The cells were either unstimulated (open bars) or stimulated with zymosan (dark blue bars) or OpZ (light blue bars) for 8 h. Extracellular media were removed and analyzed for eicosanoid levels by mass spectrometry. The data are expressed as mean values ± S.E.M. (*n* = 6). PGE2, prostaglandin E_2_; 6k-PGF1a, 6-ketoprostaglandin F_1_α; TXB2, thromboxane B_2_; dhkPGE2, 13,14-dihydro-15-ketoprostaglandin E_2_; 11-HETE, 11-hydroxy-eicosatetraenoic acid; 12-HHT, 12- hydroxyheptadecatrienoic acid; LTB4, leukotriene B_4_; LTE4, leukotriene E_4_; 5-HETE, 5-hydroxyeicosatetraenoic acid; 12-HETE, 12-hydroxyeicosatetraenoic acid; 15-HETE, 15-hydroxyeicosatetraenoic acid; tnr-12-HETE, tetranor-12-hydroxyeicosatetraenoic acid; 5,15-diHETE, 5,15-dihydroxyeicosatetraenoic acid; 11,12-DHET, 11,12-dihydroxyeicosatrienoic acid.

Next, we used LC/MS to determine the eicosanoids released into the extracellular media of macrophages when challenged with either zymosan or OpZ. The results are shown in Figure 2. Consistent with the AA release data, although zymosan stimulation generally produced lower absolute levels of eicosanoids than OpZ, the relative eicosanoid profile was very similar in both cases. PGE_2_ constituted by far the major metabolite produced under both stimulation conditions.

**Figure 3:**
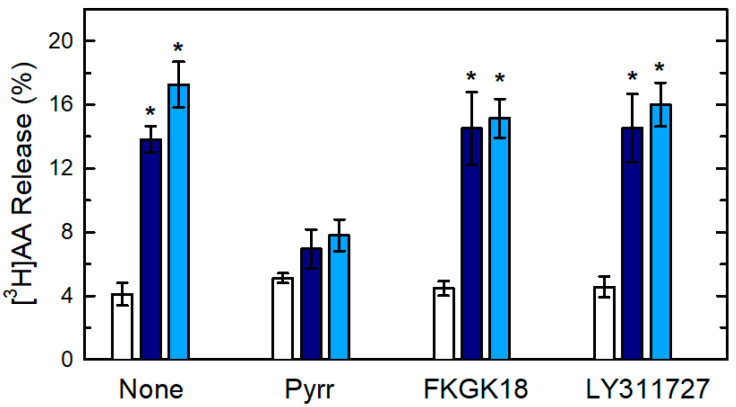
Effect of PLA_2_ inhibitors. The [^3^H]AA-labeled cells were untreated (none) or treated with 1 µM pyrrophenone (Pyrr), 10 µM FKGK18, and 25 µM LY311727 (see x-axis) for 30 min. Afterward they were untreated (open bars) or treated with 1 mg/mL zymosan (dark blue bars) or 1 mg/mL OpZ (light blue bars) for 1 h. Afterward, the extracellular media were removed and analyzed for ^3^H-radioactivity content. The data are expressed as mean values ± S.E.M. (*n* = 6). * *p* < 0.05, significance of OpZ- or zymosan-treated cells *versus* untreated cells at the indicated conditions.

To identify the PLA_2_ form(s) involved in AA release in macrophages responding to zymosan and OpZ, we took advantage of the use of the most potent and selective inhibitors currently available to block the different PLA_2_s present in macrophages [59,60]. Pyrrophenone, a cytosolic group IVA PLA_2_ (cPLA_2_α) inhibitor, is at least 3 orders of magnitude more potent for cPLA_2_α than for calcium-independent or secreted PLA_2_s [61,62,63]. The fluoroketone FKGK18 is at least 200 and 400 times more potent for calcium-independent PLA_2_ than for cPLA_2_α and secreted PLA_2_s, respectively [64]. The secreted PLA_2_ inhibitor LY311727 shows greater than 1000-fold selectivity for secreted enzymes than for cPLA_2_α or calcium-independent PLA_2_ [65]. All of these inhibitors have commonly been used to distinguish the actions of the various PLA_2_ forms present in biological systems [59,60]. Figure 3 shows that [^3^H]AA release by both zymosan and OpZ was almost completely ablated by pyrrophenone, while FKGK18 and LY311727 had no discernible effect. Collectively, these findings rule a significant role for calcium-independent or secreted PLA_2_s, and point to cPLA_2_α as the key enzyme for AA mobilization in macrophages exposed to both kinds of phagocytic stimuli. Thus, although zymosan and OpZ may activate AA release in macrophages via different receptors, the final effector of the response is the same, that is cPLA_2_α.

**Figure 4:**
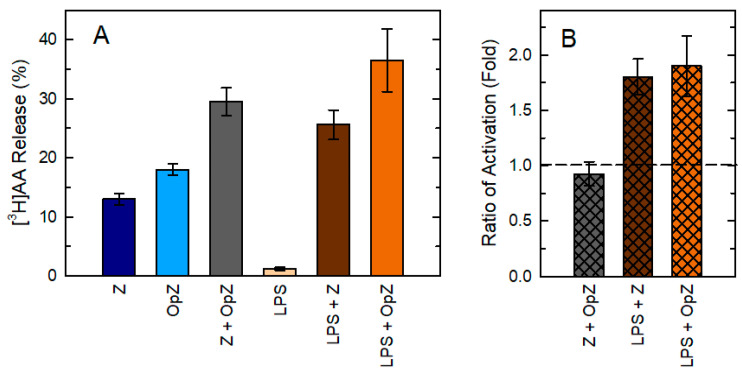
Comparison of AA release between zymosan and OpZ. (**A**) Mouse macrophages were stimulated with zymosan (0.5 mg/mL), OpZ (0.5 mg/mL) or both for 1 h, or with 100 ng/mL lipopolysaccharide (LPS) for 1 h followed by zymosan, OpZ, or neither for 1 h. Afterward, the extracellular media were removed and analyzed for ^3^H-radioactivity content. The data are expressed as mean values ± standard error of the mean of three individual measurements. (**B**) The ratio of activation of each stimulatory setting, calculated as described in the main text, is shown.

To investigate whether zymosan and OpZ stimulate cPLA_2_α through the same or divergent routes, we conducted experiments where the two agonists were added simultaneously to the cells (Figure 4). We compared the response of the two stimuli together ([Z + OpZ]) with that produced when the two stimuli were added separately ([Z] + [OpZ]). A ratio of activation was calculated as follows: Ratio of activation = [Z + OpZ]/[Z] + [OpZ]. As discussed elsewhere [66], if both stimuli acted on separate independent pathways, the AA release response would be additive, and the activation ratio would be close to 1. If the two stimuli activated the same pathway, overlapping would occur, and the combined response would be expected to be below 1. Finally, if the stimuli combined to generate more AA release than the sum of the individual responses, the ratio of activation would be greater than 1, indicating a synergistic activation. The results showed that the activation ratio when the two agonists were added together was close to 1, indicating additive effects (Figure 4). As a positive control for these experiments, we also utilized cells pretreated with bacterial lipopolysaccharide (LPS) and then stimulated with either zymosan or OpZ. Pretreatment of the macrophages with LPS is well described to enhance the AA response to a second stimulus [19,67]. As expected, the activation ratio for zymosan and OpZ in LPS-pretreated cells was well above 1.

**Figure 5:**
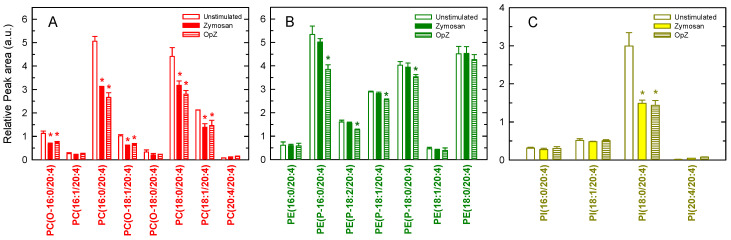
AA-containing phospholipid species in murine peritoneal macrophages and the effect of cellular stimulation. The cells were not stimulated (open bars) or stimulated with 1 mg/mL zymosan (closed bars) or 1 mg/mL OpZ (stripped bars). After 1 h, the cellular content of AA-containing choline-containing glycerophospholipid (PC) (**A**), ethanolamine-containing glycerophospholipid (PE) (**B**), and phosphatidylinositol (PI) (**C**) molecular species was determined by LC/MS. The data are expressed as mean values ± S.E.M. (*n* = 6). * *p* < 0.05, significantly different from the corresponding species in unstimulated cells. a.u., arbitray units.

Figure 5 shows the distribution of AA between the various phospholipid species of the macrophages. According to nomenclature recommendations [68], a designation of O- before the first fatty chain indicates that the sn-1 position is ether linked, whereas a P-designation indicates a plasmalogen form (sn-1 vinyl ether linkage). The fatty acyl chains are abbreviated as the number of carbon atoms: number of double bonds. The ethanolamine plasmalogens, several diacyl PCs, and inositol species PI(18:0/20:4) were detected as the major AA-containing species. Treatment of the macrophages with either zymosan or OpZ induced noticeable decreases in the PC and PI species. Strikingly, while no changes in PE species could be detected in the zymosan-stimulated cells, clear decreases in PE plasmalogen species were appreciated in the OpZ-stimulated cells (Figure 5).

**Figure 6:**
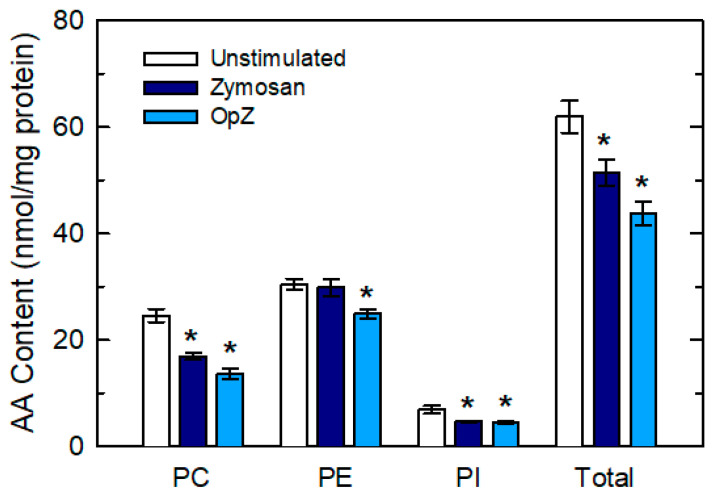
Effect of cellular stimulation on the levels of AA in different phospholipid classes. The cells were untreated (open bars) or treated with 1 mg/mL zymosan (dark blue bars) or 1 mg/mL OpZ (light blue bars) for 1 h. The AA content of the various phospholipid classes was determined by gas chromatography/mass spectrometry (GC/MS). Data are expressed as means ± S.E.M. (*n* = 6). * *p* < 0.05, significantly different from unstimulated cells.

In keeping with these data, total mass measurements of cellular AA by GC/MS indicated that mass decreases in AA-containing choline-containing glycerophospholipid (PC) and phosphatidylinositol (PI) were comparable for both zymosan and OpZ-stimulated cells; yet, decreases in AA-containing ethanolamine-containing glycerophospholipid (PE) were observed only after OpZ stimulation (Figure 6). Thus the differential utilization of AA-containing phospholipid classes in zymosan- versus OpZ-stimulated cells, demonstrates that, depending on receptor, separate mechanisms for regulating lipid turnover and free AA availability exist.

**Figure 7:**
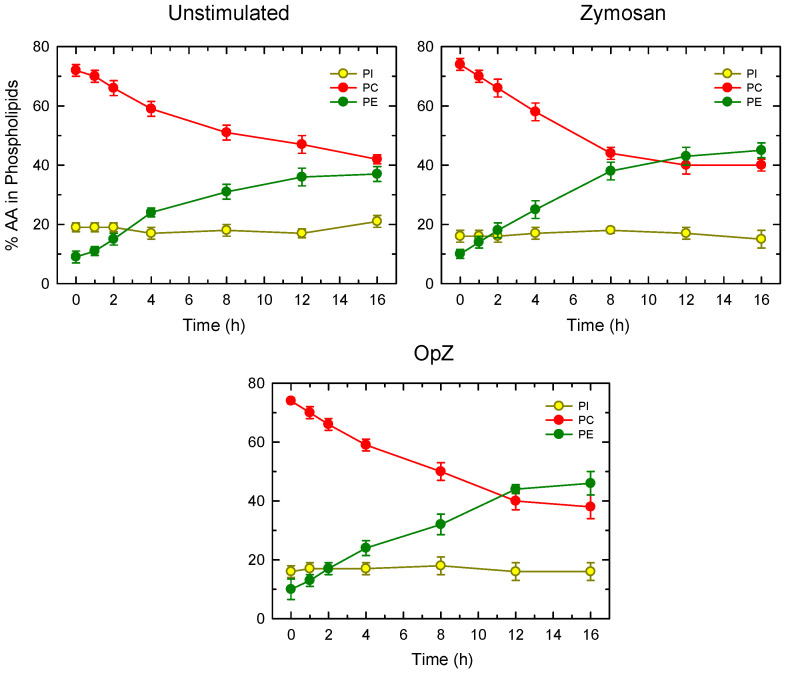
Phospholipid AA remodeling in peritoneal macrophages and the effect of cellular stimulation. The cells were pulse-labeled with [^3^H]AA, washed, and incubated without label for the indicated periods of time in the absence (unstimulated; left panel) or presence of 1 mg/mL zymosan (middle panel) or 1 mg/mL OpZ (right panel). Phospholipid classes were separated by thin-layer chromatography. The radioactivity incorporated into each phospholipid class was determined by scintillation counting and is given as a percentage of the total radioactivity present in phospholipids. Results are shown as means ± S.E.M. (*n* = 6).

The absence of AA losses from PE species in zymosan-stimulated macrophages has previously been described by us and other investigators [15,69], and it is thought to reflect the action of CoA-independent transacylase (CoA-IT), which rapidly and efficiently replenishes the AA pool in PE (both 1-acyl and 1-alkenyl species) at the expense of PC (1-acyl species) [70,71,72,73]. In analogy with the zymosan-stimulated cells, we had anticipated that, in OpZ-stimulated cells, overall AA changes in PE would also be masked by the continuing transfer of AA from PC via CoA-IT. Thus, the finding that OpZ-stimulated cells behave in a different manner was both striking and unexpected, and prompted us to directly analyze the movement of AA moieties from PC to PE via CoA-IT. For these experiments, the cells were spiked with [^3^H]AA for 30 min, thoroughly washed to remove the label that was not incorporated into phospholipids, and stimulated with either zymosan or OpZ. At different times the [^3^H]AA present in PC, PE, and PI was determined. As shown in Figure 7, PC, which constituted the major reservoir of [^3^H]AA at the beginning of the experiment, gradually experienced a time-dependent decrease in label, which was paralleled by a similar increase of [^3^H]AA in PE, reflecting the action of CoA-IT. PI species are known not to participate in CoA-IT reactions [70,71,72,73]; hence, [^3^H]AA levels in PI did not change over the time-course of the experiment. To allow for direct comparisons between different treatments, a ‘remodeling time’ was defined as the time at which the label amount in PC equals that of PE [49,74]. Figure 7 shows that, in stimulated cells, the remodeling time is >16 h, reflecting the slow nature of AA transfer between phospholipids in resting cells [70,71,72,73]. In zymosan-stimulated cells, the remodeling time was 10.6 ± 0.2 h. Interestingly, the remodeling time in OpZ-stimulated cells was noticeably higher, 11.3 ± 0.3 h, thus indicating a slower transfer of [^3^H]AA from PC to PE.

**Figure 8:**
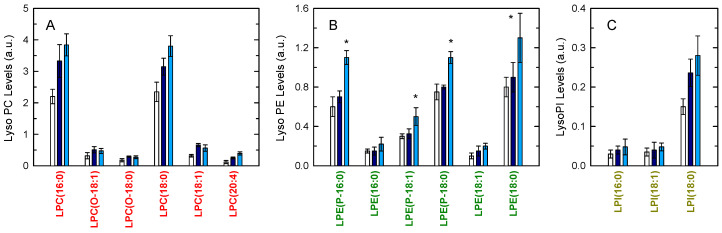
Lysophospholipid molecular species generated by macrophages and the effect of cellular stimulation. The cells were not stimulated (open bars) or were stimulated with 1 mg/mL zymosan (dark blue bars) or 1 mg/mL OpZ (light blue bars). After 1 h, the cellular content of lysoPC (**A**), lysoPE (**B**), and lysoPI (**C**) molecular species was determined by liquid chromatography/mass spectrometry (LC/MS). Results are shown as means ± S.E.M. (*n* = 6). * *p* < 0.05, significantly different from zymosan-stimulated cells. a.u., arbitray units.

On account of these data, we anticipated that, because lysoPE (both alkenacyl and diacyl forms) is the initial acceptor in the CoA-IT-mediated AA remodeling reactions, the levels of lysoPE in OpZ-stimulated cells would be elevated in comparison with those in zymosan-stimulated cells. This would be so because of the slower action of CoA-IT in the OpZ-stimulated cells. Figure 8 shows that, while lysoPC and lysoPI levels were similar under both activation regimes, the levels of several lysoPE species were higher in the OpZ-treated cells.

**Figure 9:**
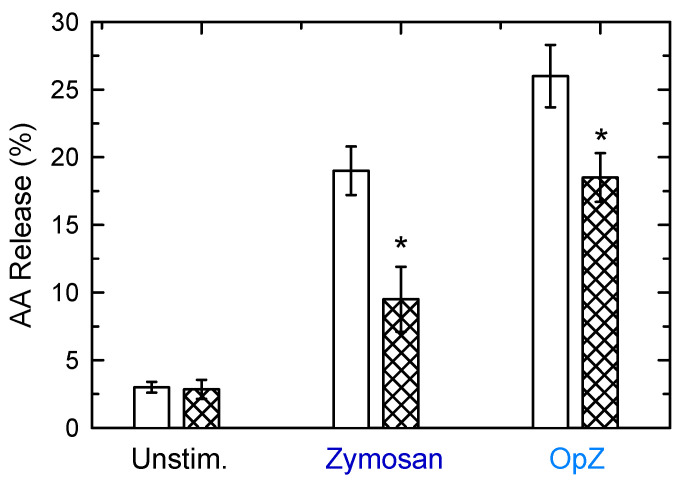
Effect of SKF98625 on macrophage AA release. The cells were left untreated (open bars) or pretreated (cross-hatched bars) with 10 µM SKF98625 for 30 min. Afterward, the cells were either untreated (unstim.) or stimulated with 1 mg/mL zymosan or 1 mg/mL OpZ (see x-axis). After 1 h, the extracellular media were removed and analyzed for 3H-radioactivity content. The data are expressed as mean values ± S.E.M. (*n* = 6). * *p* < 0.05, significantly different from the corresponding incubations without SKF9625.

CoA-IT-mediated remodeling reactions are instrumental to maintain AA in the appropriate phospholipid pools for subsequent utilization [70,71,72,73]. Thus, we investigated next whether the slower movement of AA between phospholipids in the OpZ-treated cells had any consequence on cellular function. To this end, we studied the effect of inhibiting cellular CoA-IT on macrophage AA release. We utilized the CoA-IT inhibitor SKF98625 [21,22]. This inhibitor is analogous to compounds that inhibit acyl-CoA/cholesterol acyltransferase, and has been confirmed to potently block the movement of AA from PC to PE in a variety of cells, including mouse peritoneal macrophages, with little or no effect on cellular acyl hydrolases [15,21,22,23]. Figure 9 shows that pretreating the peritoneal macrophages with SKF98625 led to strong reduction (~50%) of the AA release in response to zymosan. Importantly, the response to OpZ was also inhibited, but to a much lesser extent (~20%), thus indicating that the latter is less dependent on CoA-IT-mediated AA transfer between phospholipid species.

## 4. Discussion

Macrophages have long been recognized as major cellular sources of free AA and oxygenated metabolites. These cells contain unusually high levels of AA esterified in membrane phospholipids (20−25%) [75], and respond to a wide variety of innate stimuli by releasing the fatty acid from its phospholipid storage sites, which is efficiently metabolized to an array of oxygenated products [6,7,76]. In this study, we have comparatively investigated the AA release response of mouse peritoneal macrophages to native (zymosan) versus opsonized (OpZ) stimuli. The results of our studies demonstrate that macrophages readily respond to both stimuli by releasing free AA and eicosanoids in a time- and concentration-dependent manner, but interesting differences in overall lipid turnover exist between the two stimuli. While the overall pattern of the responses was similar, we noted that the response to OpZ was consistently higher than to zymosan. Importantly, these differences seem to correlate with an increased utilization of AA-containing PE species by the OpZ-stimulated cells compared with zymosan-stimulated cells. Application of mass spectrometry-based lipidomic analyses in identifying glycerophospholipid changes during zymosan stimulation of the macrophages indicated decreases in the levels of all major AA-containing PC species, as well as the major PI species PI(18:8/20:4). These changes were also observed in the OpZ-stimulated cells, but in this case decreases in the levels of major AA-containing PE species were also detected. Thus these data reflect a fundamental difference between the two stimuli in terms of the substrates utilized, which translate into varying cellular responses.

While AA is incorporated into various PC and PI molecular species via direct acylation reactions mediated by CoA-dependent acyltransferases [7,77], a major route for AA incorporation into PE molecular species is mediated by CoA-IT. This enzyme catalyzes the direct transfer of AA from a phospholipid donor to a lysophospholipid acceptor in the absence of CoA or ATP [70,71,72,73]. Thus, no free fatty acid intermediate is involved in the reaction. PE species (both alkenyl-acyl and diacyl) are major AA acceptors in this reaction, while AA-containing diacyl-PC is a major donor [70,71,72,73]. Although the CoA-IT sequence remains unknown, its activity has been well characterized by measuring the changes in radiolabeling of PC versus PE in cells over time, as done in the present study, and pharmacological inhibitors such as the one used in this study have been developed [21,22,78]. Previous studies in zymosan-stimulated cells showed that the AA released from PE by the action of activated PLA_2_ is replenished by the continuing action of CoA-IT, which reintegrates the AA back into PE at the expense of PC [15,19,44,49,69]. The contribution of PE to overall AA release under these conditions is manifested when experiments are conducted in the presence of CoA-IT inhibitors, which prevent refilling of PE with AA from PC [15]. Thus, the key finding that significant PE hydrolysis was detectable in the OpZ-stimulated cells, but not in the zymosan-stimulated cells even in the absence of CoA-IT inhibitors prompted us to investigate CoA-IT-dependent AA remodeling reactions under these conditions.

Macrophage stimulation with either zymosan or OpZ resulted in an increased transfer of AA moieties from PC to PE, indicating that this process is regulated by the activation state of the cells. This is in agreement with previous work in mast cells showing increased AA remodeling when the cells were exposed to different stimuli [79]. Importantly, the movement of AA from PC to PE was faster in the zymosan-stimulated cells compared with the OpZ-stimulated cells, as manifested by a shorter remodeling time. This suggests a lesser contribution of the CoA-IT remodeling pathway to phospholipid turnover in macrophages responding to OpZ. Consistent with this view is also the finding that lysoPE levels in OpZ-stimulated cells are higher than in zymosan-stimulated cells, clearly indicating that processes leading to lysoPE clearance are faster and/or more effective in the latter. Moreover, by using the CoA-IT inhibitor SKF98625, we have unveiled a key functional consequence of the AA remodeling pathway to macrophage activation. CoA-IT inhibition results in strong blockade of the zymosan response but has a comparatively much lesser effect on the OpZ response. Thus, CoA-IT mediated AA remodeling is critical for the inflammatory response to some stimuli, but not others. It is worth noting in this regard that, while the AA response to OpZ is mediated primarily by Fc receptors, it may occur that a limited number of β-glucan residues may still be exposed in the opsonin-coated particle that may contribute to the overall AA response. This could account for the smaller dependence of the OpZ response to CoA-IT inhibition. In support of this view, we have noted in preliminary experiments that stimulation of macrophages with IgG-coated latex beads leads to a cPLA_2_α-mediated AA mobilization response that is unaffected by the CoA-IT inhibitor SKF98625, suggesting no involvement of the CoA-IT pathway under these conditions. It should be kept in mind, however, that the macrophage response to IgG-coated latex beads is considerably less potent than that triggered by OpZ; hence, a more limited phospholipid turnover would be expected to occur.

Our observation that, depending on the receptor involved to activate the cPLA_2_α-dependent AA mobilization response, there is a differential involvement of CoA-IT-driven remodeling reactions is striking, and leads to a number of interesting possibilities. One is that AA is compartmentalized within the cell, such that distinct fatty acid pools are accessible to cPLA_2_α, depending on the receptor involved. Thus, AA-containing PE may represent a pool that is tightly coupled to direct hydrolysis by cPLA_2_α, but not to CoA-IT when stimulation occurs via Fc receptors. In contrast, stimulation via dectin-1 receptors would result in the AA-containing PE pool being efficiently used by CoA-IT to abstract AA from PC and, in this way, limit the production of eicosanoids that derive primarily from AA-containing PC pools [15,19,39,80]. Similarly, failure to replenish the AA-containing PE pool during stimulation with OpZ could help explain the fact that the quantities of free AA and PGE_2_ produced in OpZ-stimulated macrophages are consistently higher than in zymosan-stimulated cells because, owing to reduced AA usage for phospholipid remodeling, there is more fatty acid available in PC for lipid mediator synthesis. Collectively, these results are relevant to innate immunity and inflammation in that they identify the contrasting role of CoA-IT in limiting the amount of free AA available for production of inflammatory lipid mediators, depending on the innate receptor involved. Thus, a reduced CoA-IT rate after cell stimulation will increase the amount of free AA available for eicosanoid synthesis. Conversely, an increased rate of AA transfer by CoA-IT diverts AA to phospholipid pools and decreases its availability for eicosanoid production.

## 5. Conclusions

In addition to the expression and activity of AA-metabolizing enzymes, availability of the fatty acid in free form also constitutes a limiting step for the synthesis of eicosanoids during inflammation. Recent studies have shown that the level and composition of the eicosanoids produced may also depend upon the subcellular distribution of AA within phospholipid pools, because the AA-hydrolyzing PLA_2_ may not access all these pools equally. In innate immune cells, phospholipid AA remodeling reactions continuously occur to maintain the appropriate distribution of the fatty acid. The major enzyme involved in such AA remodeling is CoA-IT, an enzyme that moves AA directly from PC to PE pools. We have described in this work that, depending on receptor, the participation of CoA-IT to overall lipid turnover varies. Thus, rapid CoA-IT-mediated AA remodeling is observed when the cells are activated with zymosan, which activates cPLA_2_α via dectin-1 receptors. In contrast, OpZ, which activates cPLA_2_α via engagement of Fc receptors, exhibits slower remodeling of AA between membrane phospholipids and an enhanced AA mobilization and eicosanoid response. A functional consequence of these differences is that the enhanced rate of hydrolysis of PE species observed in OpZ-stimulated cells is not detected in the zymosan-stimulated cells. Overall, the results suggest that, while, PC would appear to be the major ultimate source of AA used for eicosanoid biosynthesis, the PE species may also serve as significant sources that, depending on stimulation conditions, may or may not be replenished. Collectively, these results provide information to improve our knowledge of cellular pathways regulating AA availability, which may be utilized for the design of novel strategies to interfere with eicosanoid pathways in innate immunity and inflammation. Moreover, these results may provide a better understanding of host immune responses to fungal aggression, which could help develop therapies for treating such a challenging, difficult to cure, kind of infection [81].

## Abbreviations

AA	Arachidonic acid
CoA-IT	Coenzyme A-independent transacylase
GC/MS	Gas chromatography coupled to mass spectrometry
LC/MS	Liquid chromatography coupled to mass spectrometry
OpZ	Opsonized zymosan
PC	Choline-containing glycerophospholipid
PE	Ethanolamine-containing glycerophospholipid
PI	Phosphatidylinositol
PLA_2_	Phospholipase A_2_
cPLA_2_α	Cytosolic group IVA phospholipase A_2_
iPLA_2_	Calcium-independent phospholipase A_2_
sPLA_2_	Secreted phospholipase A_2_
